# The anti-depressant effects of a novel PDE4 inhibitor derived from resveratrol

**DOI:** 10.1080/13880209.2021.1907422

**Published:** 2021-04-13

**Authors:** Yingcong Yu, Jinhui Wang, Xianfeng Huang

**Affiliations:** a Wenzhou People’s Hospital, Clinical Institute Affiliated to Wenzhou Medical University, Wenzhou, PR China; b School of Pharmacy & School of Medicine, Changzhou University, Changzhou, PR China

**Keywords:** Polyphenol, behaviour test, depression

## Abstract

**Context:**

Resveratrol has shown anti-stress and anti-depressant-like abilities involved in inhibiting phosphodiesterase-4 (PDE4) enzyme. However, its application is limited due to its low efficacy, bioavailability and selectivity.

**Objective:**

This study synthesized a new resveratrol derivative RES003 and evaluated its PDE4 inhibitory and anti-depressant-like activities *in vitro* and *in vivo*, respectively.

**Materials and methods:**

PDEs inhibitory activities were evaluated by radioactive tracer method. Anti-depressant-like activities of novel resveratrol analogue (RES003) at doses of 2.5, 5.0 and 10 mg/kg was investigated by sugar water consumption and forced swimming tests using male ICR mice under chronic unpredictable stress procedure for 10 days. A total of 84 mice were randomly distributed into seven groups (*n* = 12). Drugs and vehicle were administered (intra-gastric or intra-peritoneal) once a day from the first to the last day. The molecular mechanisms were identified by western blot.

**Results:**

RES003 showed more potent PDE4 inhibitory activity (half maximal inhibitory concentration (IC_50_), 0.87 μM) and better selectivity than resveratrol (IC_50_, 18.8 μM). RES003 could significantly increase the consumption of sugar water (*p* < 0.01) and immobility time (*p* < 0.01) compared to vehicle-treated stressed groups at doses of 5 and 10 mg/kg. Furthermore, RES003 could significantly increase the levels of cyclic adenosine monophosphate response element binding protein phosphorylation (10 mg/kg, *p* < 0.05) and brain-derived neurotrophic factor (BDNF) expression (5 and 10 mg/kg, *p* < 0.05 and 0.01) in mouse brain.

**Discussion and conclusions:**

RES003 could ameliorate chronic stress induced depression-like behaviours through inhibition of PDE4 and activation of cAMP-triggered phosphorylation of cAMP response element binding protein/BDNF signalling pathway. Consequently, RES003 is a promising lead compound for the treatment of depression.

## Introduction

Depression is a serious mental disorder that may cause morbidity and mortality in children and adolescents. It is characterized by high rates of suicide, anxiety or irritable mood along with cognitive and sleep disorder (Schoepf et al. 2014; Silove et al. 2015). Depressed patients are usually reflected by changes in brain monoamine neurotransmitters, specifically noradrenaline (norepinephrine [NE]) and 5-hydroxytryptamine ([5-HT], serotonin). In fact, currently available anti-depressant drugs such as tricyclic anti-depressants, selective serotonin reuptake inhibitors, monoamine oxidase inhibitors and serotonin and noradrenaline reuptake inhibitor are formulated based on 5-HT or NE function in the brain. However, only one-third of individuals with depression or anxiety show full remission in response to these medications (Zhang et al. 2014). Thus, development of novel anti-depressants with different mechanisms of action is urgent.

Resveratrol, a natural polyphenol being present in many plant-based foods, has many pharmacological properties including anti-oxidant and anti-inflammatory activities (Tredici et al. 1999; Chen et al. 2007; Ranney and Petro 2009). Recent studies suggested that resveratrol could alleviate depression-like symptoms induced by stress in rats by non-specifically inhibiting cyclic adenosine monophosphate (cAMP) phosphodiesterases (PDEs) such as PDE3 and PDE4 (Park et al. 2012; Wang et al. 2016). Furthermore, several phenolic compounds structurally related to resveratrol were proved to be selective PDE4 inhibitors. PDEs, being subclassified into 11 different families (PDE1-11), have proven to be druggable targets, however, achieving high selectivity among the gene family can be challenging and is desirable for adequate safety profiles since unwanted PDE inhibition has been reported to be associated with side effects (Young and Ward 1988; Cote et al. 2003). PDE4 has been implicated in anti-depressant and anxiolytic activities by catalyzing the hydrolysis of cAMP, which plays a critical role in the cellular response to extracellular stimuli (Zhao et al. 2013). Therefore, resveratrol may be an attractive agent for the treatment of depression by inhibiting PDE4 and regulation of cAMP level in the brain.

The usefulness of resveratrol, however, is limited by its chemical instability and low bioavailability (Baur and Sinclair 2006; Kang et al. 2009; Coimbra et al. 2011). Recently, considerable attention has been focussed on *E*-resveratrol derivatives. In an effort to overcome these problems and enhance the pharmacological activity of resveratrol, several groups have attempted to synthesize resveratrol derivatives. Some of these compounds were reported to exhibit strong neuroprotective, pro-apoptotic and anti-proliferative effects (Belluti et al. 2010; Fulda 2010; Mazue et al. 2010; Biasutto et al. 2014, 2017).

**Figure 1.**
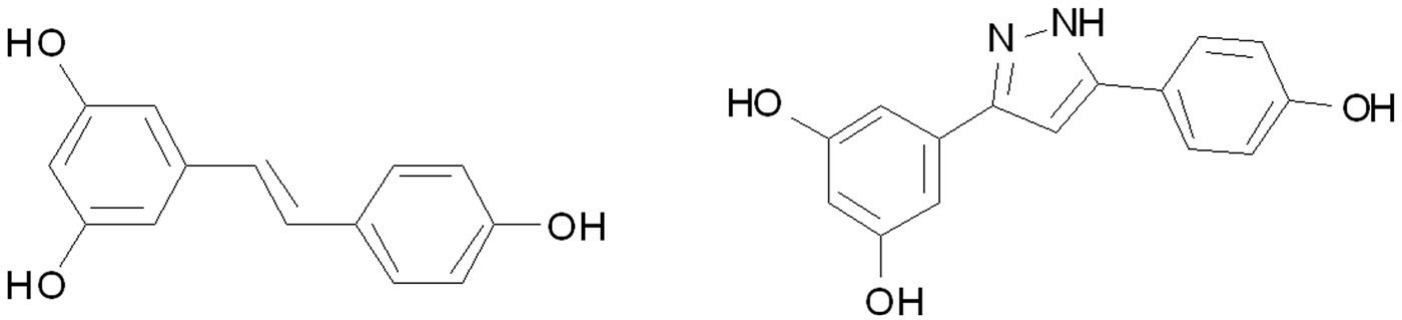
The structures of resveratrol and RES003.

Encouraged by the promising activity of the reported *E*-resveratrol derivatives, we constructed a novel resveratrol analogue (RES003) by introducing a pyrazole group between the two phenyl rings (Figure 1). Its anti-depressant-like effect was evaluated both *in vitro* and *in vivo*. Our results indicated that the resveratrol analogue may be developed as a leading compound for novel anti-depressant agents.

## Materials and methods

### Reagents

All chemical reagents (analytical grade) were purchased from Shanghai Aladdin Bio-Chem Technology Co., LTD (Shanghai, China), and used as received without any purification. Reactions were monitored by thin layer chromatography (TLC) using Merck silica gel 60 F254 pre-coated plates (0.25 mm) and column chromatography was performed with silica gel (100–200 mesh). All products were characterized by proton nuclear magnetic resonance (^1^H NMR) and mass spectroscopy (MS). ^1^H NMR spectra was acquired on a Bruker DRX 400 NMR spectrometer (Bruker, Billerica, MA) using dimethylsufone as solvent. MS spectra were acquired on a Thermo Scientific LTQ Orbitrap XL mass spectrometer (Thermo Scientific, Waltham, MA).

### Synthesis of RES003

#### Procedure for preparation of compounds **3** and **4**

Compounds **1** [1-(3,5-dihydroxyphenyl)ethanone, 1 mmol] or **2** (4-hydroxybenzaldehyde, 1 mmol), 3,4-dihydro-2H-pyran (1 or 2 mmol) and pyridinium *p*-toluenesulfonate salt (PPTs, 0.05 mmol) were added to dichloromethane (CH_2_Cl_2_, 50 mL) in a round bottom and the reaction mixture was stirred at room temperature for 8–10 h. When the reaction was completed (monitored by TLC), the reaction mixture was poured into 10% sodium hydroxide (NaOH) solution (50 mL), and the resulted mixture was extracted twice with 50 mL of CH_2_Cl_2_. The organic layer was dried over magnesium sulfate (MgSO_4_) followed by removal of the solvent under reduced pressure to afford the compounds **3** (291 mg, yield: 90.9%) and **4** (196 mg, yield: 95.1%).

1-[3,5-bis(Tetrahydro-2H-pyran-2-yloxy)phenyl]ethanone (**3**): ^1^H NMR (400 MHz, dimethyl sulfoxide [DMSO]) δ 7.26 (s, 2H), 6.93 (s, 1H), 5.58 (s, 2H), 3.81 (q, 3H), 3.62 (t, 2H), 2.55 (s, 3H), 1.98-1.90 (m, 4H), 1.60-1.52 (m, 4H), 1.52-1.48 (m, 4H); electrospray ionization (ESI)-MS: 321.2 [M + 1]^+^.

4-(Tetrahydro-2H-pyran-2-yloxy)benzaldehyde (**4**): ^1^H NMR (400 MHz, DMSO) δ 9.87 (s, 1H), 7.70 (d, 2H, *J* = 8.1 Hz), 7.10 (d, 2H, *J* = 8.1 Hz), 5.60 (s, 1H), 3.78-3.60 (m, 2H), 1.98 (t, 2H), 1.62-1.56 (m, 2H), 1.72-1.65 (m, 2H); ESI-MS: 207.1 [M + 1]^+^.

#### Procedure for preparation of compound **5**

Compounds **3** (1.0 mmol) and **4** (1.0 mmol) were dissolved in 100 mL of dry methyl alcohol (CH_3_OH), followed by dropping of 4% NaOH solution (8 mL) and white precipitate was formed. The mixture was then refluxed for 24 h. The obtained precipitate was filtered, and dissolved in ethanol (C_2_H_5_OH, 8 mL), then 0.5 mmol pyridinium *p*-toluenesulfonate salt was added. The mixture was heated to 50 °C for 24 h. The solvent was removed under vacuum, the residue was partitioned between ether and water. The organic layer was then dried over MgSO_4_ and concentrated under reduced pressure. Flash chromatography [Ethyl acetate (EtOAc)/petroleum ether] furnished compound **5** (161 mg, yield: 62.9%).

(*E*)-1-(3,5-Dihydroxyphenyl)-3-(4-hydroxyphenyl)prop-2-en-1-one (**5**): ^1^H NMR (400 MHz, DMSO) δ 10.10 (s, 1H), 9.56 (s, 2H), 7.80 (d, 2H, *J* = 8.7 Hz), 7.79-7.49 (m, 2H), 6.70-7.02 (m, 4H), 6.49 (s, 1H); ESI-MS: 257.6 [M + 1]^+^.

#### Procedure for preparation of compound RES003

Compound **5** (1 mmol) and hydrazine hydrate (1 mmol) was added to C_2_H_5_OH (100 mL) in a round bottom, followed by addition of several drops of acetic acid (CH_3_COOH). The mixture was refluxed for 48 h. The solvent was removed under vacuum, the residue was partitioned between EtOAc and water. The organic layer was then dried over MgSO_4_ and concentrated under reduced pressure. Flash chromatography (EtOAc/petroleum ether) furnished compound RES003 (218 mg, yield: 81.3%).

5-[5-(4-Hydroxyphenyl)-1H-pyrazol-3-yl]benzene-1,3-diol (RES003): ^1^H NMR (400 MHz, DMSO) δ 9.87 (s, 1H), 9.58 (s, 1H), 9.29 (s, 2H), 7.62 (d, 2H, *J* = 8.0 Hz), 6.80 (m, 3H), 6.66 (m, 2H), 6.19 (s, 1H). ^13^C NMR (125 MHz, DMSO) δ 162.68, 160.13, 158.81, 145.90 (2 C), 134.22, 129.77, 128.02 (2 C), 114.32 (2 C), 106.19, 105.22, 102.37, 97.19. HR-MS (*m/z*) (ESI): calcd. for C_15_H_13_N_2_O_3_ [M + H]^+^: 269.0926, calcd. 269.0989.

#### Enzymatic assay

All the enzymatic activities of PDEs were assayed by using ^3^H- cyclic guanosine monophosphate (cGMP) or ^3^H-cAMP as the substrate. PDEs were firstly incubated with the reaction mixture of 50 mmol/L Tris-hydrogen chloride (HCl), pH 7.8, 10 mmol/L magnesium chloride (MgCl_2_) and ^3^H-cAMP or ^3^H-cGMP (40, 000 cpm/assay) at 24 °C for 30 min. Then, the reaction was terminated by addition of zinc sulfate and barium hydroxide. Activity of test compounds was assessed by measuring the amount of ^3^H-cGMP or ^3^H-cAMP resulting from enzyme cleavage. The reaction product ^3^H-AMP or ^3^H-GMP was precipitated by adding barium sulfate, while unreacted ^3^H-cAMP or ^3^H-cGMP remained in the supernatant. Radioactivity in the supernatant was measured in 2.5 mL of ultima gold liquid scintillation. At least eight concentrations of inhibitors (0.05–500 μM) were used in the presence of suitable substrates for the measurement of half maximal inhibitory concentration IC_50_ values, which were calculated by non-linear regression. Each measurement was repeated three times.

#### Animals, chemicals and drug treatments

Male ICR mice, weighing between 22–25 g, were obtained from the Animal Centre at Changzhou University. All experiments were carried out according to the National Institute of Health Guide for Care and Use of Laboratory Animals (1996) and were approved by the Institutional Animal Care and Use Committee of Changzhou University (NO. Y20180023). Fluoxetine and resveratrol were purchased from Sigma Chemical Co. (St Louis, MO). For animal behavioural tests, RES003 and rolipram were dissolved in saline containing 5% DMSO, 5% Solutol and 90% saline. The positive control drug fluoxetine was dissolved simply in saline. Mice were given intra-gastric (i.g.) administration (RES003: 2.5, 5.0 and 10 mg/kg) or intraperitoneal (i.p.) injections (fluoxetine: 5 mg/kg and rolipram: 10 mg/kg) in a volume of 10 mL/kg body weight.

### Rolipram

The chronic unpredictable stress procedure was carried out as described previously with minor modification (Polster and Fiskum 2004). Before beginning the chronic unpredictable stress, mice were housed individually for one day and subjected to chronic unpredictable stress for 10 days. Then the animals were exposed to two different stressors each day, which include 2 h restraint, damp bedding, tilted cage, 10 min forced-forced swim and overnight illumination. All procedures were performed in isolated rooms adjacent to the housing room. Control mice were individually housed for the same period and were handled daily for 30 s in the housing room without being stressed.

After the chronic un-predictable stress (CUS) procedure, sucrose preference test was assessed on the following day, and then forced swimming test (FST) was carried out the next day. Mice were treated with vehicle, RES003 and fluoxetine 30 min before being subjected to the sucrose preference test (SPT) and FST. Drugs and vehicle were administered (i.g. or i.p.) once a day from the first day and continued until the last day. After behavioural testing, all the animals were used for biochemical analysis.

### Behavioural tests

#### SPT

The SPT was performed according to the previous description with minor modification (Yu et al. 2013). Before the procedure, animals were food and water deprived for 14 h. Then, mice were exposed to both the test solution (1% sucrose) and water for a 24 h period during the test. The sucrose preference was calculated as the ratio of the consumed sucrose solution to the total amount of liquid consumed.

#### FST

According to the previous description, animals were individually placed in glass cylinders (25 cm height, 10 cm diameter) containing 10 cm depth of water at 24 ± 1 °C for 6 min, allowing for free swimming (Porsolt et al. 1978). There was a 15 min pre-test followed 24 h later by a 5 min test. A mouse was determined to be immobile when there were only small movements necessary to keep its head above water. The immobility time was recorded during the 5 min testing period.

#### Locomotor activity (LA) test

LA test was assessed using an open-field chamber according to previous procedure (Li et al. 2014). The floor of the chamber was divided into 16 identical squares. Mice were individually placed in the centre of the chamber and allowed to acclimatize for 15 min. Their paws were contacted or disconnected the active bars which can produce random configurations. The locomotor counts of mice were measured by counting the number of line crossings in the following 10 min.

#### Western blotting

The brain tissues were dissected and stored at −80 °C until analysis after mice were sacrificed. All the tissues were homogenized in ice-cold RIPA buffer containing 0.1% phenylmethylsulfonyl fluoride and centrifuged at 14,000 rpm for 20 min at 4 °C. The supernatant was taken to assess cAMP-triggered phosphorylation of cAMP response element binding protein (pCREB), cAMP-response element binding protein (CREB)and brain-derived neurotrophic factor (BDNF) protein expression. Samples (50 mg protein each) were separated using sodium dodecyl sulphate-polyacrylamide gel electrophoresis before transferring to nitrocellulose membranes, non-specific bindings were blocked in blocking buffer for 2 h at room temperature. After washing, membranes were subsequently incubated with rabbit anti-phosphorylated CREB (1:1000; Millipore, Billerica, MA), rabbit anti-CREB (1:1000; Millipore), rabbit anti-BDNF (1:1000; Abcam, HongKong) and rabbit anti-β-actin antibodies (1:1000; Cell signalling Technology, Boston, MA) at 4 °C overnight. The membranes were then incubated with goat anti-rabbit IgG-HRP (1:10,000; Millipore, CA) for 60 min. The detection and quantification of specific bands were performed using a fluorescence scanner and ‘Image-Pro’ software. Band stripping was carried out by incubating the membranes with stripping buffer for 15 min.

### Statistical analysis

Data shown were expressed as mean ± standard error of the mean (SEM). Repeated measures analysis of variance (ANOVA) and multi-variate ANOVA were used to analyze consumption of sugar water and immobility time in the behavioural tests. Other data were analyzed by one-way ANOVA followed by the Newman-Keuls comparison test. *p* < 0.05 was considered statistically significant.

### Docking simulations

Molecular docking of resveratrol and RES003 into the three-dimensional X-ray structure of PDE4 (PDB code: 4WCU) was carried out using CDOCKER protocol of Discovery Studio 2019 (BIOVIA, France).

## Results

### Synthesis of RES003

**Scheme 1.**
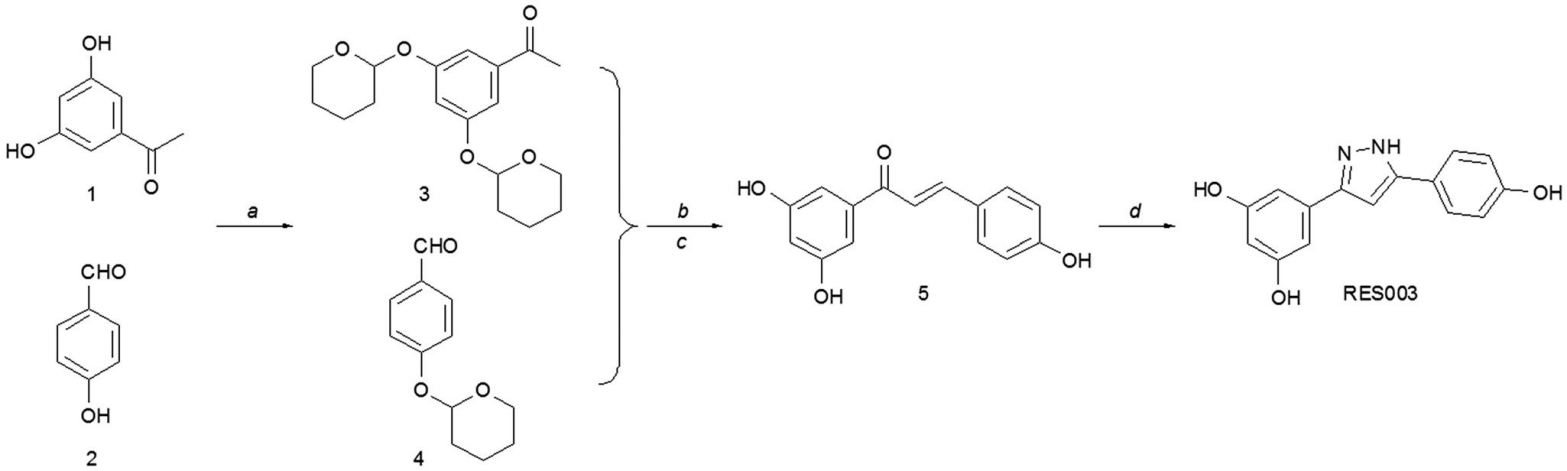
Synthesis of RES003. Reagents and conditions: (a) PPTs, CH_2_Cl_2_, room temperature; (b) NaOH, CH_3_OH, reflux; (c) PPTs, C_2_H_5_OH, 50 °Cand (d) hydrazine hydrate, CH_3_COOH, C_2_H_5_OH, reflux.

The synthesis of RES003 was carried out by a multi-step procedure according to Scheme 1. In the first step, the hydoxyl groups of the commercially available phenols **1** and **2** were protected using 3,4-dihydro-2H-pyran and yielded intermediates **3** and **4**. Subsequently, compounds **3** and **4** were condensed in the presence of sodium hydroxide to give **5**. After the removal of the protecting group, the intermediate **6** reacted with hydrazine hydrate to afford to RES003.

### Evaluation of PDE4 inhibitory activity, selectivity and docking

**Figure 2.**
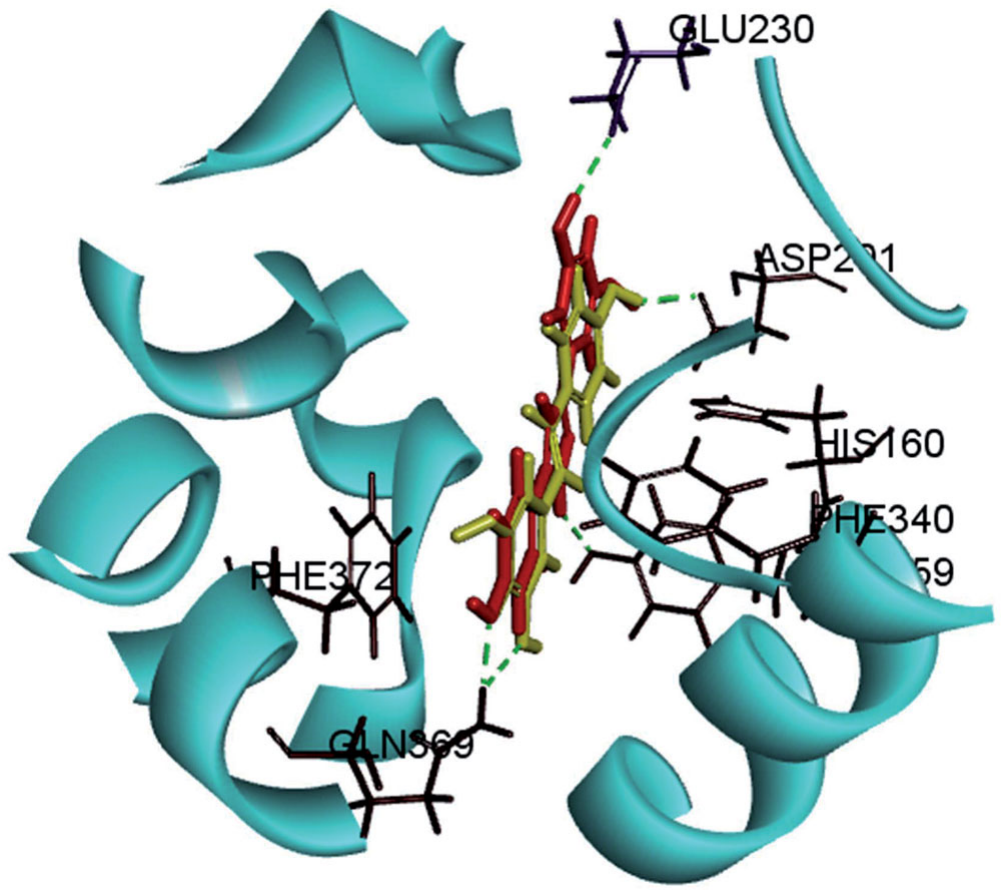
Binding mode of compound resveratrol and RES003 within the active pocket of PDE4 (PDB ID: 1UDU). Hydrogen bonds are represented by dashed lines.

Bioassay validations for the RES003 and resveratrol were performed. RES003 showed a high PDE2 selectivity with a IC_50_ value of 0.87 ± 0.11 μM against PDE4 and displayed > 100-fold over PDE1 (92.6 ± 8.3 μM), PDE2 (>100 μM), PDE3 (>100 μM) and PDE5 (>100 μM), respectively, revealing that it was poor active against other PDE family members. The predicted binding mode of compound RES003 within the active site pockets of PDE4 is presented in Figure 2. It showed RES003 can make contact with PDE4 strongly via hydrophobic interactions, face-to-face π-π stacking interactions and hydrogen bonds.

### The anti-depressant-like effects of RES003 in SPT and FST

**Figure 3.**
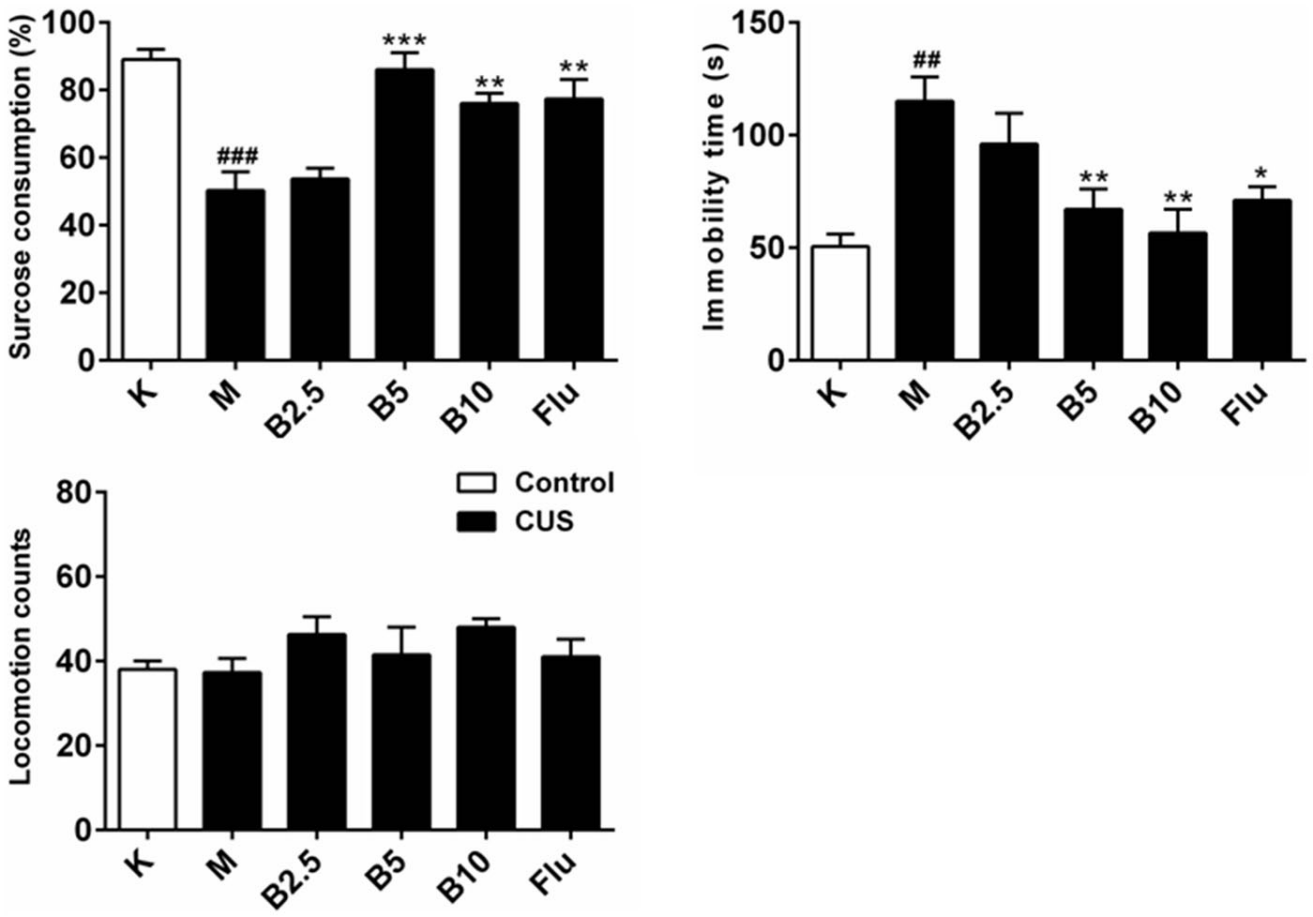
The anti-depressant-like effects of RES003 in mice. Mice were treated with vehicle, RES003, and fluoxetine 30 min before being subjected to the (A) SPT and (B) FST. (C) Mice were treated with various doses of RES003 30 min before the locomotor activity test. Results are expressed as mean ± SEM (*n* = 9–10). ^##^*p* < 0.05 and ^###^*p* < 0.001 versus vehicle-treated control group. *p < 0.05 and **p < 0.01 versus model group. K: control; M : CUS model; Flu: fluoxetine.

As we can see from Figure 3(A), compared with the blank group, the consumption of sugar water was significantly reduced in the model group (*p* < 0.001). Compared with the model group, both the large and medium dose RES003 groups could significantly increase the consumption of sugar water in chronic stress depression rat models [F_4,32_ = 12.53, *p* < 0.01]. Flu group exhibited similar effects at doses of 5 mg/kg. As shown in Figure 3(B), chronic unpredictable stress induced a significant increase in immobility time in forced swimming tests (*p* < 0.01). Administering RES003 at a dose of 5 and 10 mg/kg (i.p.) significantly reduced immobility time, when compared to vehicle-treated stressed groups [F_4,40_ = 10.21, *p* < 0.01]. Mice treated with Flu also exhibited significant anti-depressant-like effects (*p* < 0.05).

The RES003 treatment at the dose that significantly reduced immobility response did not affect the locomotor activity as shown in Figure 3(C), indicating the anti-depressant-like effects of RES003 were not due to central stimulation or inhibition. As expected, RES003 produced significant anti-depressant effects.

### The effects of RES003 on pCREB/CREB and BDNF expression in mouse brain

**Figure 4.**
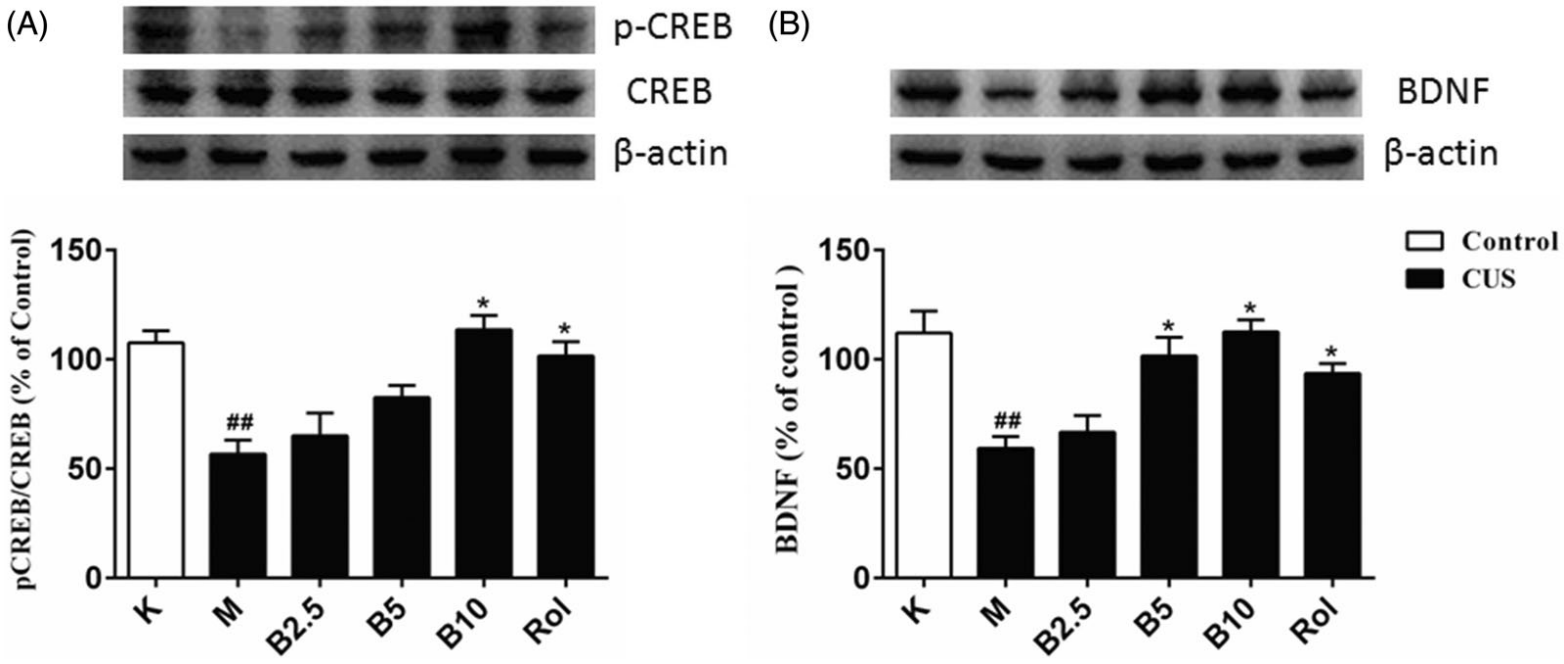
Effects of RES003 on CUS-induced decreases in the ratio of pCREB/CREB and BDNF expression in the brain tissue. Results are expressed as mean ± SEM (*n* = 9–10). ^##^*p* < 0.05 versus vehicle-treated control group. *p < 0.05 versus model group. Rol: rolipram.

To determine whether PDE4 inhibition by RES003 upregulated downstream CREB phosphorylation and BDNF expression, we analyzed the ratio of pCREB/CREB and BDNF levels in mouse brain. As shown in Figure 4, CUS exposure significantly decreased the ratio of pCREB to CREB and BDNF in the brain tissue of CUS mice when compared to control groups (*p* < 0.01, p < 0.01). However, the decreased pCREB/CREB was reversed by RES003 at dose of 10 mg/kg (*p* < 0.05). BDNF expression was also significantly upregulated at doses of 5 and 10 mg/kg (*p* < 0.05 and 0.01). As the positive drug, similar effects were observed when treatment with rolipram at 10 mg/kg (*p* < 0.05).

## Discussion

PDE4 is a novel and valuable pharmacological target in the central nervous system (CNS). Its characteristics of hydrolyzation of cyclic nucleotides and selective distribution in forebrain structures make it critical in controlling several CNS disorders including depression, anxiety, and learning and memory disabilities. Resveratrol could alleviate depression-like symptoms induced by stress in mice by inhibiting PDE4, but it is non-specific. In this study, the experimental IC_50_ values of resveratrol against PDE4, PDE1, PDE2, PDE3 and PDE5 were 18.8 ± 2.7, 8.7 ± 1.1, >100, 12.7 ± 2.1 and >100 μM, respectively, which are consistent with the previously reported results (Park et al. 2012). Clearly, when the double bond of resveratrol was replaced by pyrazole ring, the PDE4 inhibitory activities were increased significantly (IC_50_ for RES003: 0.67 μM). Furthermore, RES003 showed better selectivity over PDE1, PDE 2, PDE 3 and PDE 5 compared with resveratrol. To explore the interaction mode of RES003 with PDE4, molecular docking simulation was performed using discovery studio 2019 software based on the crystal structure of PDE4 complexed tadalafil (PDB ID: 1UDU). The predicted binding mode of compound RES003 within the active site pockets of PDE4 showed the compound contacts with Phe340, Phe372 of PDE4 via hydrophobic interactions, which make important contributions to the binding between PDE4 and its inhibitors. It is noticeable that the pyrazole ring also interacts with histamine160 through a face-to-face π-π stacking interaction. Furthermore, three hydroxyl groups and a amine group could form four hydrogen bonds with Gln369, Glu230, Asp201 and Tyr159, respectively. By contrast, though RES was accommodated into the active pocket with a similar behaviour, its double bond does not interact with PDE4. It is clear that the synthesized RES003 binds to PDE4D more strongly than RES.

Behavioural studies play an important role in the evaluation of anti-depressant drugs. To detect if compound RES003 has physiological effects *in vivo*, we evaluated the behavioural changes of chronic stress-induced mice using sucrose preference and forced swimming tests. Our results demonstrated that RES003 was effective in both sucrose preference and forced swimming tests. Both tests are sensitive to anti-depressant treatment thus providing validation for drug efficacy. Furthermore, RES003 did not affect the locomotor activity at doses that produced anti-depressant-like effects, indicating its specific anti-depressant-like effects.

The findings that RES003 increased pCREB and subsequent BDNF expression in the brain of CUS mice suggested that the PDE4 inhibitor RES003-induced neuroprotective effects were probably mediated by cAMP or cGMP-dependent pCREB/CREB and BDNF expression.

In the signalling pathways involving PDEs, PKA and PKG regulate the expression of key genes in central nervous system by activating some transcription factors (Menniti et al. 2006; Kleppisch 2009). CREB is one of the transcription factors mainly activated by phosphorylation on the serine 133 residue (Shaywitz and Greenberg 1999). And the major transcriptional outcome of CREB phosphorylation/activation is thought to be the production of BDNF. In the mice hippocampus and other brain regions, BDNF also regulates nerve cell plasticity and development, which may be achieved through promoting cell survival and reducing neuronal damage (Takahashi et al. 1999; Banasr et al. 2004). The findings that RES003 increased pCREB and subsequent BDNF expression in the brain of CUS mice suggested that the PDE4 inhibitor RES003-induced neuroprotective effects were probably mediated by cAMP or cGMP-dependent pCREB/CREB and BDNF expression.

## Conclusions

Taken together, the present study showed the synthesis of a novel PDE4 inhibitor RES003 and selectivity upon PDE4. The *in vivo* study suggested RES003 can ameliorate chronic stress induced depression-like behaviours through inhibition of PDE4 and activation of pCREB/BDNF signalling pathway. This study provides evidence that a novel PDE4 inhibitor deriving from resveratrol may be useful for the treatment of depressive disorders.
